# Seroprevalence of hepatitis c virus infection among blood donors in Ethiopia: a systematic review and meta-analysis

**DOI:** 10.1186/s12879-021-05827-z

**Published:** 2021-01-30

**Authors:** Eyasu Alem Lake, Robera Olana Fite, Lielt Gebreselassie Gebrekirstos, Meron Hadis Gebremedhin, Mohammed Suleiman Obsa, Kelemu Abebe Gelaw

**Affiliations:** 1 Department of Nursing, College of Health Science And Medicine, Wolaita Sodo University, Wolaita Sodo, Ethiopia; 2 School of Medicine, College of Health Science and Medicine, Wolaita Sodo University, Wolaita Sodo, Ethiopia; 3 Department of Anesthesia, College of Health Science and Medicine, Wolaita Sodo University, Wolaita Sodo, Ethiopia; 4 Department of Midwifery, College of Health Science and Medicine, Wolaita Sodo University, Wolaita Sodo, Ethiopia

**Keywords:** Seroprevalence, Hepatitis C, Blood donors, Ethiopia

## Abstract

**Background:**

Blood transfusion is one of the routine therapeutic interventions in hospitals that can be lifesaving. However, this intervention is related to several transfusion-related infections. Hepatitis C viral infection is one of the most common causes of transfusion-related hepatitis. Subsequently, this systematic review and meta-analysis was aimed to estimate the seroprevalence of hepatitis C virus infection among blood donors in Ethiopia.

**Methods:**

PubMed, Google Scholar, Health InterNetwork Access to Research Initiative (HINARI), Excerpta Medica database (EMBASE), and Cochrane library, the web of science, African journal of online (AJOL), and Google Scholar was searched. The data were extracted using Microsoft Excel and analyzed by using STATA version 14. Publication bias was checked by funnel plot, contour-enhanced funnel plots**,** trim and fill analysis and more objectively through Egger’s regression test, with *P* <  0.05 considered to indicate potential publication bias. The heterogeneity of studies was checked using I2 statistics. Pooled analysis was conducted using a weighted inverse variance random-effects model. Subgroup analysis was done by region and study period. A sensitivity analysis was employed.

**Result:**

A total of 25 studies with 197,172 study participants were used to estimate the seroprevalence of hepatitis c virus among blood donors. The overall seroprevalence of hepatitis C virus was 0.819% (95% CI: 0.67–0.969; I2 = 92.3%). Regional sub-group analysis showed that the pooled prevalence of hepatitis c virus infection among blood donors found to be 0.563% in Somali, 1.08% in Oromia, 0.847% in Amhara, and 0.908% in south nations nationalities and peoples region.

**Conclusion:**

The pooled seroprevalence of hepatitis C virus infection among blood donors in Ethiopia found to be low. Moreover, there should be systematic strategies that enhance donor screening and retention of safe regular donors.

## Background

Blood transfusion is a critical retroactive intercession that encompasses a basic part of patient management [1]. The World Health Organization (WHO), to assure the quality and safety, recommends screening of donated blood for a minimum of the major transfusion-transmittable infections, including human immunodeficiency virus (HIV), hepatitis B virus (HBV), hepatitis C virus (HCV) and syphilis [2]. Ethiopian national blood bank was set up in 1969. It is one of a kind open benefit that has been working to support the blood donation for the last half-century within the nation. Annually Ethiopian Red Cross distributes 42,000 units of collected and screened blood to various health institutions in the country [3]. However, access to safe blood and blood products remains a major challenge throughout the world, especially in developing countries. In Africa alone, up to 500 peoples acquire transfusion-transmissible infections (TTIs) due to contaminated blood transfusion daily [4]. Unless the donated blood screened systematically, each transfused patient will have a chance of getting transfusion-transmittable diseases particularly HIV, hepatitis B virus, hepatitis C virus and syphilis [2].

Hepatitis C virus (HCV) which has been placed in the family of *Flaviviridae* was found in 1989 [5]. It is one of the common cause of transfusion-related hepatitis [6]. The transmission of HCV happens essentially through the introduction to contaminated blood during blood transfusion, organ transplantation, intravenous medicate utilization, body piercings, hemodialysis, and occupational exposure [7]. After infection, HCV generally persisted in the host leading to a chronic infection in 75 to 85% of cases [7, 8].

The worldwide seroprevalence of HCV estimated to be 2.5% ranges from 2.9% in Africa and 1.3% in America and the prevalence among blood donors varies from 0.4 to 19.2%. The estimated chance of HCV transmission during blood transfuses ranges from 0.10 to 2.33 per million units transfused [9, 10]. According to WHO estimation, in 2015 about 71 million people infected with hepatitis C virus and a significant number of cases continue to an incessant carrier which is at risk for liver cirrhosis and cancer [11, 12]. In Africa nationwide estimation of HCV is limited and those studies are usually outdated. A systematic review and meta-analysis estimation of HCV among African adults was conducted by taking studies published from 2000 to 2014 and the result showed that the seroprevalence of HCV among blood donors was found to be 0.65% [13].

WHO is working to improve access to health care for all and it introduced a comprehensive package for the vaccination, diagnosis and treatment of viral hepatitis including HCV [14]. The sustainable development goal 3.3 sets to addresses a range of challenging communicable diseases including hepatitis that interfere with normal growth and development [15]. To achieve this WHO recommends that each country should work towards self-sufficiency of safe blood and blood products aiming a 100% donations from regular, voluntary and non-remunerated blood donors [14, 15].

Ethiopia is amongst the higher affected area by liver disease which accounts for 12% hospital admission and 31% mortality at inpatient departments of Ethiopian health institutions [16]. As far as our knowledge, there are limited studies on HCV seroprevalence among blood donors in Ethiopia and those presented studies showed that there is a significant difference in the magnitude of HCV among various regions. The variation also exists within a specific region.

Although there has been a competent vaccine in Ethiopia since 2007 that target hepatitis B vaccine which given on the 6th, 10th and 14th weeks of birth, there has been no known vaccine targeting HCV [17]. A study by Shiferaw and his colleagues revealed that a key informant from the federal ministry of health (FMOH) believed HCV is low in prevalence due to improved infection-control procedures in the health institution and blood banks. But no written evidence exposed what kind of intervention done in the country [18].

A systematic review and meta-analysis of hepatitis viral infection was conducted in 2016 by taking studies done from 1968 to 2015 and the pooled prevalence of HCV was found to be 3.1% [19]. However, this study didn’t provide a clear and comprehensive estimation on the seroprevalence of HCV among blood donors in Ethiopia. This study stood with a footnote, a study which articulates the national seroprevalence of HCV among blood donors in Ethiopia is essential. Therefore, this study was conducted to provide the estimated seroprevalence of the problem in the nation as input for program planning, realizing and evaluating interventions that are focused on the prevention and control of transfusion-transmittable infections.

## Methods

### Searching strategy and source of information

This study was conducted to estimates the pooled prevalence of hepatitis C virus infection among blood donors in Ethiopia. The presence of the existing systematic review and meta-analysis was checked using the DARE database (http://www.library.UCSF.edu) and the Cochrane library to avoid duplication. We also checked the availability of any similar ongoing review to the current systematic review and meta-analysis in the PROSPERO database ((PROSPERO 2017:CRD42017074407); Available from http:// www.Crd.york.ac.uk/ PROSPERO_REBRANDING/ display record. Asp? ID = CRD42017074407and we decided that there was no previous similar study to the topic.

All relevant and published researches in the following databases; PubMed, Google Scholar, Excerpta Medica database (EMBASE), CINHAL, Cochrane library, Web of Science, and African journal of online (AJOL) was searched. We salvaged grey literature using Google. Unpublished studies were taken from the official website of an international and local organization or university.

The following core search terms or phrases were used; Seroprevalence, prevalence, hepatitis C, hepatitis C virus, HCV, Transfusion-transmissible infection, blood donors, blood transfusion, and volunteer donors. Search terms were pre-defined to allow a complete search strategy that included all-important studies. All fields within records and MeSH (Medical Subject Headings) and Boolean operators were used to search in advance PubMed search engine.

Notably, to fit advanced PubMed database the following search strategy were developed using different Boolean operators; ((((((Blood donors [tw] OR blood transfusion [tw] OR volunteer donors [tw])) OR (“Blood Transfusion, Intrauterine”[Mesh] OR “Blood Transfusion, Autologous”[Mesh] OR “Blood Transfusion”[Mesh] OR “Blood Donors”[Mesh] OR “Transfusion Reaction”[Mesh] OR “Platelet Transfusion”[Mesh] OR “Blood Component Transfusion”[Mesh]))) AND (((Seroprevalence [tw] OR prevalence [tw])) OR (“Prevalence”[Mesh] OR “HIV Seroprevalence”[Mesh] OR “Contraceptive Prevalence Surveys”[Mesh] OR “Leukocyte Nuclear Appendages, Hereditary Prevalence of” [Supplementary Concept] OR “Seroepidemiologic Studies”[Mesh] OR “epidemiology” [Subheading] OR “Cross-Sectional Studies”[Mesh]))) AND (((5. Hepatitis c [tw] OR Hepatitis C virus [tw] OR HCV [tw] OR Transfusion-transmissible infection* [tw])) OR (“Hepatitis C Antibodies”[Mesh] OR “Hepatitis C, Chronic”[Mesh] OR “Hepatitis C Antigens”[Mesh] OR “Hepatitis C”[Mesh] OR “NS-5 protein, hepatitis C virus” [Supplementary Concept] OR “hepatitis C protein F, Hepatitis C virus” [Supplementary Concept] OR “core protein p22, Hepatitis C virus” [Supplementary Concept] OR “glycoprotein E2, Hepatitis C virus” [Supplementary Concept] OR “E1 protein, Hepatitis C virus” [Supplementary Concept] OR “glycoprotein 35, Hepatitis C virus” [Supplementary Concept] OR “nucleocapsid protein, Hepatitis C virus” [Supplementary Concept] OR “core protein p13, Hepatitis C virus” [Supplementary Concept] OR “core protein p9, Hepatitis C virus” [Supplementary Concept] OR “p7 protein, Hepatitis C virus” [Supplementary Concept] OR “proteinase Cpro-2, hepatitis C virus” [Supplementary Concept] OR “NS4A cofactor peptide, Hepatitis C virus” [Supplementary Concept] OR “21 kDa protein, hepatitis C virus” [Supplementary Concept] OR “16 kDa protein, hepatitis C virus” [Supplementary Concept] OR “NS3-4A serine protease, Hepatitis C virus” [Supplementary Concept] OR “HCV 796” [Supplementary Concept] OR “core protein (1-120), hepatitis C virus” [Supplementary Concept] OR “hepatitis C immune globulin, human” [Supplementary Concept] OR “(5-cyano-8-methyl-1-propyl-1,3,4,9-tetrahydropyrano (3,4-b)indol-1-yl) acetic acid” [Supplementary Concept] OR “PCLAF protein, human” [Supplementary Concept] OR “NS4 protein, hepatitis C virus” [Supplementary Concept] OR “Y19 proten, Hepatitis C virus” [Supplementary Concept]))) AND Ethiopia [tw].

### Reporting

The results of this review were reported based on the Preferred Reporting Items for Systematic Review and Meta-Analysis statement (PRISMA) guideline [20].

### Eligibility criteria

#### Study type

- All observational study

#### Study content

- A study that reported about seroprevalence or Prevalence of hepatitis C viral infection using a valid HCV screening test.
- Prevalence is also calculated using the data presented in the studies.

#### Study period

- There was no restriction on the study period

#### Blood donor type

- There was no restriction on the type of blood donors

#### Exclusion criteria

1. Interventional studies, a study conducted on non-humans
2. Reviewed articles
3. Studies reporting confused data or probable errors
4. Studies without any information on the country were exempted from the reviewing
5. Studies which were not fully accessed an attempt was done to contact the corresponding author using the email address or mobile phone written in the published articles

### Study selection and extraction

Retrieved articles were exported to the reference manager software, Mendeley Desktop to remove duplicate studies. Three independent reviewers screened the title and abstract. The disagreement was handled based on established article selection criteria. Data were extracted using a standardized data extraction format prepared in Microsoft Excel by two independent authors. Any discrepancy during extraction was solved through discussion. The name of the first author, study area and region, the study period, the study design, year of publication, study population, sample size, response rate and prevalence of HCV were collected.

### Quality assessment

Three independent authors appraised the quality of the studies. The Joanna Briggs Institute (JBI) quality appraisal checklist was used [21]. When there is any disagreement all the three authors discussed and resolved it. The critical appraisal checklist has 8 parameters with yes, no, unclear and not applicable option. The parameter involves the following questions: (1) Were the criteria for inclusion in the sample clearly defined?, (2) Were the study subjects and the setting described in detail?, (3) Was the exposure measured in a valid and reliable way?, (4) Were objective, standard criteria used for measurement of the condition?, (5) Were confounding factors identified?, (6) Were strategies to deal with confounding factors stated?, (7 Were the outcomes measured in a valid and reliable way?, and (8) Was appropriate statistical analysis used?. Studies were considered low risk when it scored 50% and above of the quality assessment indicators.

### Statistical analysis

The data were extracted using Microsoft Excel and analyzed by using STATA version 14 statistical software (stataCorp LP, 4905 Lakeway Drive, College Station, TX 77845, USA). Publication bias was checked by funnel plot and more objectively through Begg and Egger’s regression tests, with *P* <  0.05 considered to indicate potential publication bias [22]. Using Duval and Tweedie recommendation a trim and fill analysis (Metatrim) was done to see the effect of publication bias [23]. It adds studies to make the distribution symmetrical. The presence of significant between-study heterogeneity was assessed using cochrane Q statistic. I^2^ was used to quantify between-study heterogeneity, in which a value of 0, 25, 50, and 75% represented no, low, medium, and increased heterogeneity, respectively [24]. A forest plot was used to visualize the presence of heterogeneity. Since we found a high level of heterogeneity, we used a random-effect model for analysis to estimate Der Simonian and Laird’s pooled effect. Subgroup analysis was done by region and study period. A leave-one-out sensitivity analysis was employed to see the effect of a single study on the overall meta-analysis estimate. The result was presented in the form of text, table, and figures.

## Result

### Search outcomes

**Fig. 1:**
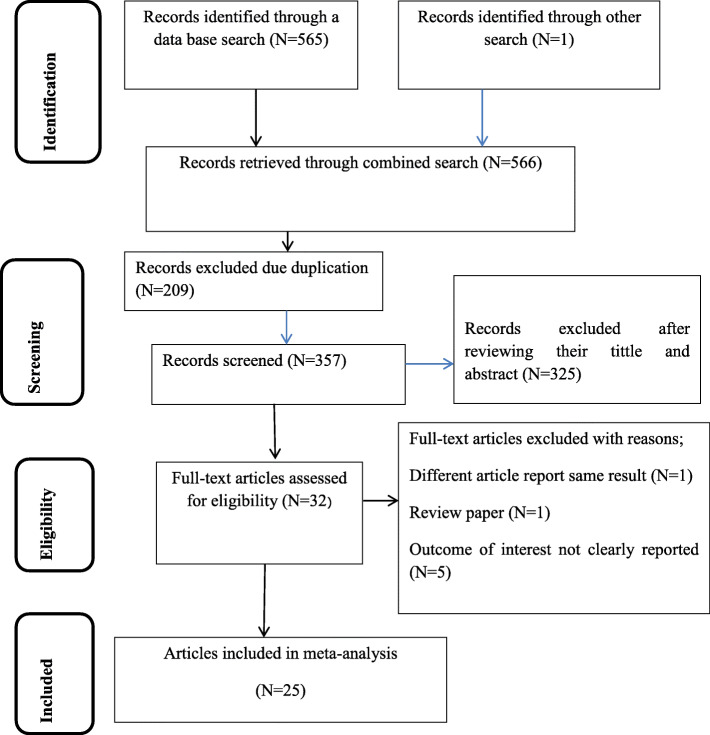
Schematic presentation of study selection for systematic review and meta-analysis of HCV seroprevalence among blood donors in Ethiopia

At first, 566 articles were retrieved using electronic search. Of these articles, 209 were excluded due to duplication and 32 articles were fully accessed and assessed for qualification. Eventually, 25 articles met the eligibility criteria and were included in the final meta-analysis (Fig. 1).

### Characteristics of included studies

Twenty-five articles, which fulfilled the inclusion criteria, were included in this systematic review and meta-analysis. All studies had a low risk during the quality assessment. Twenty-two articles concerning the seroprevalence of hepatitis c virus infection were obtained from seven regions. Nine studies were conducted in Amhara region [25–33], six in south nations nationalities and Peoples (SNNP) [34–39], two in Oromia [40, 41], two in Somali [42, 43], one in Tigray [44], one in Dire Dawa [45], one in Harari [46] and three studies area were not restricted by regions since there study area was more than one region including the capital, Addis Ababa [47–49].

The earliest study was conducted by Gelaw and Mengistu in 2003 at Amhara and Tigray region. The recent studies were four studies that were conducted in 2018. Regarding the study design, 24 studies employed a cross-sectional study design (retrospective or prospective type) and one study was conducted through a retrospective cohort. The sample size ranges from 148 to 5885. All the studies included in this systematic review and meta-analysis had a 100% rate except a single study done in Debre Tabor, Amhara region which had a 91.7% response rate. The highest seroprevalence of hepatitis c virus (13.3%) was noted in a study from Amhara region and the lowest (0.2%) was exported from a study conducted in Oromia region (Table 1].

**Table 1:** Characteristics of studies included in the systematic review and meta-analysis on hepatitis C virus seroprevalence among blood donors in Ethiopia

***Authors name***	Sampling year	Publication year	Study area	Study region	Study design	Sample size	Prevalence % (95%ci)	Logit transformation	Study quality
**Melese** ***A.*** **et al*/ ***[42]	2014	2016	Jijiga	Somali	Cross-sectional	6827	0.73	−4.927207	Low risk
**Alemeshet Y. et al **[40]	2010	2011	Jimma	Oromia	Cross-sectional	6063	0.2	−6.21661	Low risk
**Shiferaw et al** [26]	2018	2019	Bahirdar	Amhara	Cross-sectional	35,435	0.6	−5.122014	Low risk
**Bekele Sharew et al **[27]	2012	2015	Dessie	Amhara	Cross-sectional	8908	0.61	−5.105585	Low risk
**Misganew B. **[35]	2013	2016	Hawassa	SNNP	Cross-sectional	6337	0.6	−5.122014	Low risk
**Dessie et al** [28]	2006	2007	Bahirdar	Amhara	Cross-sectional	324	13.3	−2.160122	Low risk
**Tigabu et al.** [29]	2018	2019	Gondar	Amhara	Cross-sectional	5983	1.6	−4.151296	Low risk
**Negash et al.** [30]	2018	2019	Debrtabor	Amhara	Cross-sectional	310	4.2	−3.212993	Low risk
**Bonja et al.** [38]	2015	2017	Hawassa	SNNP	Cross-sectional	384	0.5	−5.30333	Low risk
**Deressa et al.** [31]	2017	2018	North Shewa	Amhara	Cross-sectional	8460	0.32	−5.747809	Low risk
**Ambachew et al** [36]	2016	2018	Hawassa	SNNP	Cross-sectional	2237	0.5	−5.30333	Low risk
**Teklemariam et al.** [46]	2017	2018	Harrer	Harari	Cross-sectional	11,382	0.8	−4.836346	Low risk
**Zerihun A. et al **[45]	2013	2018	Dire Dawa	Dire Dawa	Cross-sectional	6376	0.96	−4.655639	Low risk
**Mohammed and Bekele** [43]	2013	2016	Jijiga	Somali	Cross-sectional	4224	0.4	−5.525469	Low risk
**Tessema et al** [33]	2007	2010	Gondar	Amhara	Cross-sectional	6361	0.7	−4.96887	Low risk
**Fithamlak S. **[39]	2015	2016	W/Sodo	SNNP	Cross-sectional	390	8.5	−2.553935	Low risk
**DEGEFA ET AL.** [44]	2014	2018	Mekelle	Tigray	Cross-sectional	10,728	1.33	−4.333381	Low risk
**Biadgo et al** [32]	2012	2017	North Gondar	Amhara	Retrospective Cohort	6471	0.8	−4.836346	Low risk
**Jose M. et al **[41]	2012	2016	Gambo Hospital	Oromia	Cross-sectional	2606	2	−3.932226	Low risk
**HUNDIE ET AL** [49]	2014	2017	Nationwide	Unspecific^a^	Cross-sectional	56,885	0.52	−5.26431	Low risk
**Abate et al. **[25]	2008	2013	Bahir Dar	Amhara	Cross-sectional	2384	0.63	−5.073525	Low risk
**Heyredin et al** [48]	2018	2019	Eastern Ethiopia	Unspecific^a^	Cross-sectional	500	1	−4.615221	Low risk
**Mengistu Hailemariam **[37]	2015	2018	Hawassa	SNNP	Cross-sectional	6849	0.7	−4.96887	Low risk
**Gelaw and Mengistu** [47]	2003	2008	Amhara & Tigray	Unspecific^a^	Cross-sectional	600	1.7	−4.091688	Low risk
**Tigistu Demisse **[34]	2014	Unpublished	W/Sodo	SNNP	Cross-sectional	148	8.8	−2.522534	Low risk

^a^The study was area was in more than one region; *P* prevalence

### Publication bias

**Fig. 2:**
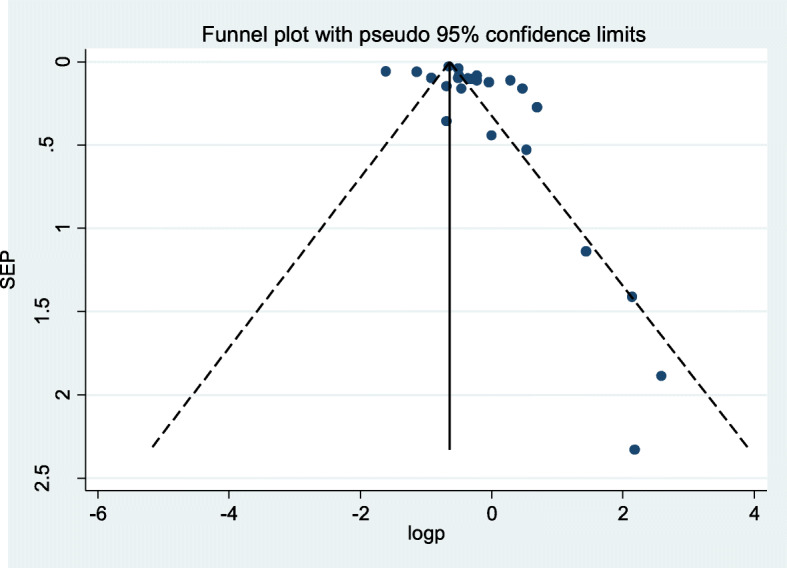
Funnel plots for publication bias of seroprevalence of hepatitis C virus infection

The presence of publication bias was assessed using funnel plot, Egger and Begg regression test at 5% significant level. There was statistical evidence of publication bias. A funnel plot showed some asymmetrical distribution, the Begg and Egger tests were statistically significant with a *p*-value = 0.002 and *p*-value = 0.004 respectively (Fig. 2).

**Fig. 3:**
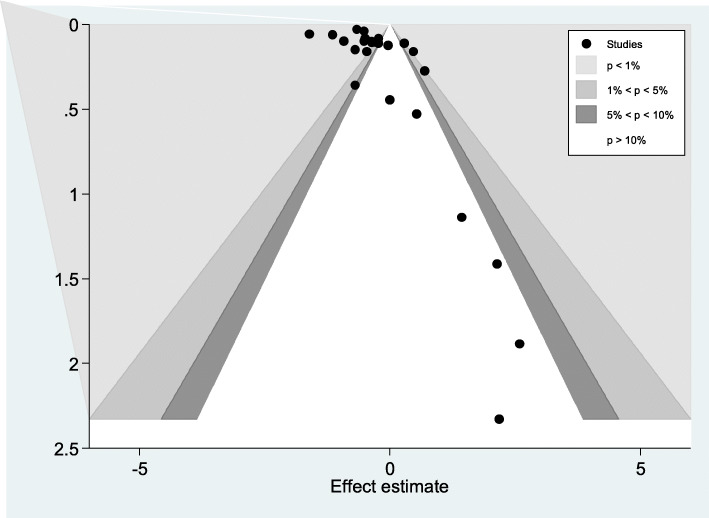
Contour-enhanced funnel plots publication bias of seroprevalence of hepatitis C virus infection

The funnel plot was asymmetric and showed a small study effect. The counter-enhanced funnel plot makes a difference in us to recognize between publication bias and other causes of asymmetry. It showed that small studies were found not only in the area of statistical significance (shaded area) but also in the areas of non-statistical significance (white area). So the asymmetry may have been caused by several factors and not exclusively by publication bias (Fig. 3).

### Trim and fill analysis (metatrim)

**Fig. 4:**
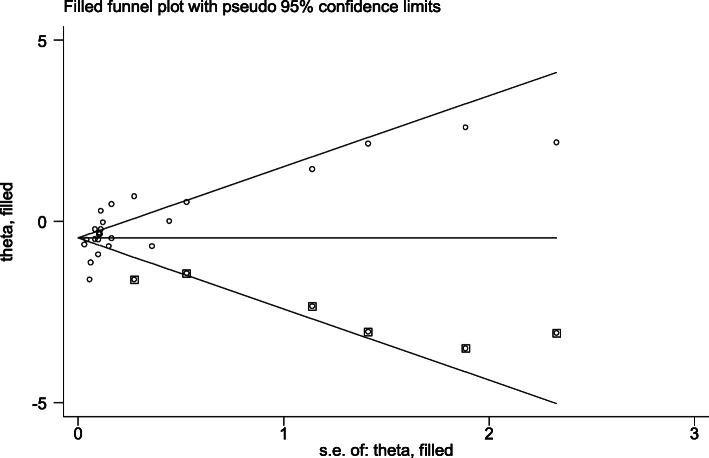
Filled funnel plots for publication bias of seroprevalence of hepatitis C virus among blood donors in Ethiopia

Egger’s regression test p-value of the study was 0.004, which demonstrated the presence of publication bias. So we conducted trim and fill analysis to see the impact of publication bias though the assumption that the funnel plot asymmetry was solely caused by publication bias might do not hold for this dataset. The trim and fill analysis showed the presence of six unpublished studies. Considering these studies in calculating the pooled prevalence yields an estimated pooled seroprevalence of HCV, which is adjusted for publication bias was found to be 0.789% (95% CI: 0.614, 0.945; *p* = 0.000) (Fig. 4).

### Seroprevalence of hepatitis c virus infection

**Fig. 5:**
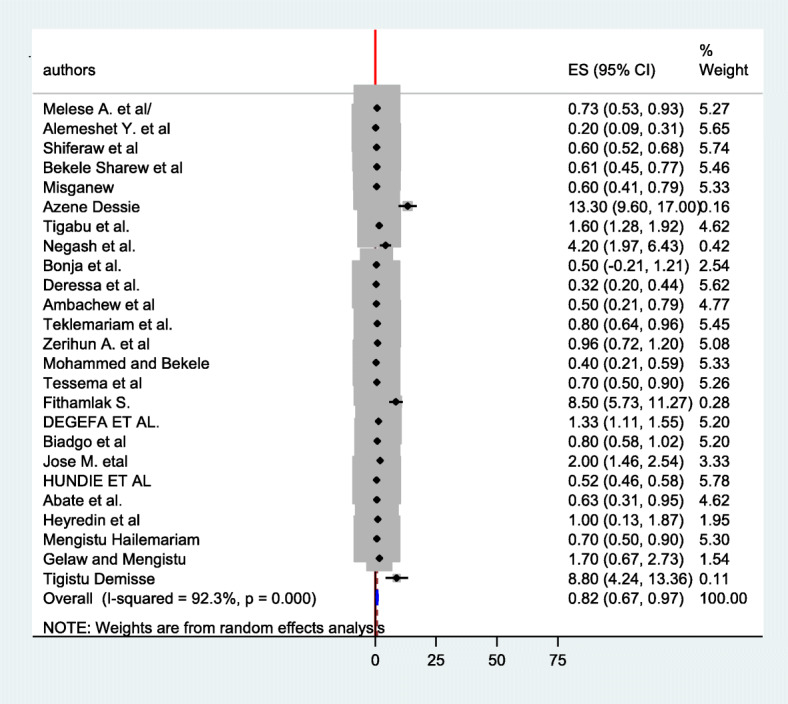
Forest plot of the seroprevalence of hepatitis C virus infection among blood donors in Ethiopia

The estimated overall seroprevalence of hepatitis c virus infection in Ethiopia presented in a forest plot (Fig. 5). Using random-effect model, the overall seroprevalence was found to be 0.819% (95% CI: 0.67–0.969; I2 = 92.3%). The finding showed that there is evidence of high statistical heterogeneity of outcome effect (I^2^ = 92.3%). To stabilize the raw prevalence estimates from each study a logit transformation was done and the pooled estimate of hepatitis c virus among blood donors in Ethiopia using logit transformation result of each proportion found to be − 4.610(95%CI: − 4.840, − 4.381; I2 = 100%).

### A leave-out-one sensitivity analysis

A leave-out-one sensitivity analysis was done to evaluate the effect of each study on the pooled seroprevalence of HCV among blood donors by excluding each study step-by-step. The result showed that the excluded study didn’t bring significant change on the estimated seroprevalence of HCV among blood donors (Table 2).

**Table 2:** A-leave-out -one sensitivity analysis for seroprevalence of hepatitis C virus among blood donors in Ethiopia

Study omitted	Pooled estimate	95%CI
**Melese A. et al/ **[42]	0.827	0.671–0.983
**Alemeshet Y. et al **[40]	0.851	0.701–1.002
**Shiferaw et al** [26]	0.853	0.684–1.023
**Bekele Sharew et al** [27]	0.837	0.679–0.995
**Misganew **[35]	0.836	0.679 0.992
**Dessie et al** [28]	0.788	0.649–0.928
**Tigabu et al.** [29]	0.771	0.626–0.915
**Negash et al.** [30]	0.803	0.655–0.950
**Bonja et al.** [38]	0.828	0.676–0.980
**Deressa et al.** [31]	0.852	0.696–1.009
**Ambachew et al** [36]	0.837	0.682–0.991
**Teklemariam et al.** [46]	0.823	0.667–0.979
**Zerihun A. et al **[45]	0.811	0.658–0.964
**Mohammed and Bekele** [43]	0.846	0.690–1.002
**Tessema et al** [33]	0.829	0.673–0.985
**Fithamlak S. **[39]	0.790	0.648–0.933
**DEGEFA ET AL.** [44]	0.779	0.634–0.923
**Biadgo et al** [32]	0.822	0.667–0.977
**Jose M. etal **[41]	0.772	0.626–0.918
**HUNDIE ET AL** [49]	0.868	0.691–1.045
**Abate et al. **[25]	0.830	0.676–0.984
**Heyredin et al** [48]	0.816	0.665–0.967
**Mengistu Hailemariam **[37]	0.829	0.673–0.985
**Gelaw and Mengistu** [47]	0.805	0.655–0.954
**Tigistu Demisse **[34]	0.808	0.661–0.955

### Subgroup analysis

The subgroup analysis based on the region and year of publication revealed that the seroprevalence of hepatitis c virus infection among blood donors was found to be 0.563% in Somali, 1.08% in Oromia, 0.847% in Amhara and 0.908% in south nation nationalities and peoples region (SNNP). According to a subgroup analysis by the year of publication, the seroprevalence of hepatitis c virus among blood donors was found to be 7.359% in the studies published before the 2010 year (Table 3).

**Table 3:** The pooled seroprevalence of hepatitis C virus infection, 95% CI and heterogeneity estimate with a *p*-value for subgroup analysis

Variable	Characteristics	Pooled seroprevalence 95%(CI)	I^**2**^(***p***-value)
**Region**	Somali	0.563%(0.240, 0.887)	81.6%(0.02)
	Oromia	1.080%(−0.684–2.844)	97.6%(< 0.001)
	Amhara	0.847%(0.576, 1.117)	93.2%(< 0.001)
	SNNP	0.908%(0.408, 1.408)	88.8%(< 0.001)
**By year of publication**	< 2010	7.359%(−4.006, 18.723)	97.1%(< 0.001)
	2010–2014	0.497%(0.124, 0.87)	90.5%(< 0.001)
	2015–2019	0.814%(0.667, 0.96)	91.1%(< 0.001)

## Discussion

Hepatitis c virus is one of the common serious transfusion-transmittable contaminations which require genuine consideration during blood transfusion. Avoidance of this viral infection in developed nations accomplished by avoiding unnecessary transfusions, using regular blood donors, barring donors with specific risk factors and orderly screening all sorts of blood that will be given. Be that as it may, in developing countries none of such meditations applied consistently and the chance of transfusion transmittable infections remains high [50]. Epidemiological studies in different regions of the world showed a wide variation in the global prevalence of hepatitis c virus with a high frequency in low-income countries [51].

In this study, we estimated the pooled seroprevalence of hepatitis c virus among blood donors by taking 25 observational studies done in the nation irrespective of blood donor type. Considering this, HCV prevalence was ranged from 0.2 to 13.3% and around 28% of study results were above 1.5% in which the finding was in line with the most affected regions [52]. In this review, the overall seroprevalence of hepatitis c virus among blood donors found to be 0.819% [0.67–0.969]. The finding was lower than a previous estimate of HCV among the general population in Ethiopia which was 3.1% [19]. The finding of this study was more or less comparable to a finding from in Middle Eastern countries which were 0.88% [51]. However, the finding was lower than those previous finding in Congo 2.7% and Chinese mainland 8.68% [53, 54]. On the other hand, the result of this study was slightly higher those previous estimates in adult African and Iran which was 0.65 and 0.5% respectively [13, 55]. Moreover, the finding of this study was within the range of the global prevalence of HCV among blood donors [9, 10].

The distinction in seroprevalence of hepatitis c virus infection among studies might be clarified due to differences in sort, educational status, and level of awareness among blood donors. Besides, study design and period and also strategies of blood testing have a considerable significance for the observed difference. Furthermore, the low sample size in some studies included in this systematic review and meta-analysis might influence the gotten results.

In this study, the highest seroprevalence of hepatitis c among blood donors was exported at Amhara region and the lowest in Oromia region [28, 40]. The high prevalence in Amhara region might be due to more than half of the study populations were not safe regular and voluntary blood donors and that might affect the overall pooled estimate. Whereas, the low prevalence in Oromia region might be because most of the donors were volunteers who might have a good awareness of HCV disease. The difference in sample population might also have a credit for the observed variation.

A regional estimate of hepatitis c virus in the sub-group analysis showed that a lower prevalence was in Somali and higher was in Oromia region. The conceivable reason for this variation could be due to sociocultural, literacy, ethnic and religious difference across regions. On the other hand, most of the studies included in this meta-analysis were conducted in urban blood donation centers while in Oromia region there’s one study which was conducted in the rural hospital and transfusion-transmittable infection among rural blood donators might be high since rural donors are usually illiterate and might have low-level of awareness about such infections.

The study period estimated of the hepatitis c virus infection in the sub-group analysis showed that the prevalence of hepatitis c virus was 7.359% in the studies published before 2010, 0.497% in 2010–2014, and 0.814% in studies published from 2014 to 2019. Studies published before 2010 had a higher seroprevalence of hepatitis c virus compared to those published after 2010. There could be a commendable change in the type of safe regular, voluntary and non-remunerated blood donors. Moreover, awareness creation and attitudinal health education on the universal precaution about hepatitis virus including the introduction of hepatitis vaccines for risky groups in Ethiopia was done though this was not consistent and this might take its claim credit for this change.

In this study, the variation between studies resulted in a significant between-study heterogeneity. To manage it we used a random-effect model. Even if, there was no influence on overall estimate a leave one-out-one sensitivity analysis was done. The result showed that the estimated pooled seroprevalence was robust and not dependent on a single study. We also tried to assess the possible source variability by sub-group analysis using study region and period. The high heterogeneity might be due to differences in the sample population between studies. Type of blood donors, screening method, study period, and paper quality might contribute to the heterogeneity.

### Strength and limitation of the study

- This study used a comprehensive searching strategy and looked at both published and unpublished studies through the different datasets to estimate the national seroprevalence of hepatitis C virus.
- More than one assessor was used in the quality evaluation and appraisal process using JBI-MAStARI.
- It might lack national representativeness since no information was found from Afar, Gambella, and Benishangul-Gumuz regions.

## Conclusion

Considering potential limitations that might arise from the addition of unpublished study, small sample size study effects along with methodological heterogeneity, the finding of this study showed that the pooled seroprevalence of HCV among blood donors in Ethiopia was found to be low. Since a considerable amount of studies included in this review, it might be valuable for policymakers and other stakeholders. Therefore, this study recommends a customary and systematized screening of donated blood using more sensitive screening instruments and enlistment of safe regular donors to preserve patients or individuals who receive blood from transfusion-transmittable viral infections. This study also recommended further research involving those regions in which study was not found and the possible contributing factors of HCV among blood donors.

## Data Availability

All data about this study are contained and presented in this document.

## Abbreviations

CI: Confidence Interval
DARE: Database of Abstracts of Review of Effects
EMBASE: Excerpta Medica database
FMOH: Federal Ministry of Health
HBV: Hepatitis B Virus
HCV: Hepatitis C Virus
HINARI: Health InterNetwork Access to Research Initiative
HIV: Human Immunodefiency virus
JBI: The Joanna Briggs Institute
PRISMA: Preferred Reporting Items for Systematic Review and Meta-Analysis
SNNP: South Nations Nationalities and Peoples Region; WHO: World Health Organization
